# Hemophagocytic lymphohistiocytosis responding to withdrawal of gluten: a case report

**DOI:** 10.1186/s13256-016-1049-6

**Published:** 2016-09-22

**Authors:** Nicholas J. Fordham, Richa Ajitsaria, Leena Karnik, Subarna Chakravorty

**Affiliations:** 1 St Helier’s Hospital, Wrythe Lane, Carshalton, Surrey SM5 1AA UK; 2 Hillingdon Hospital, Pield Heath Road, Uxbridge, UB8 3NN UK; 3 St Mary’s Hospital, Praed Street, London, W2 1NY UK; 4 Imperial College London, London, SW7 2AZ UK

**Keywords:** HLH, Hemophagocytosis, Coeliac disease, Gluten

## Abstract

**Background:**

This is the first documented case of a patient with hemophagocytic lymphohistiocytosis in association with coeliac disease. There was complete clinical and biochemical remission of hemophagocytic lymphohistiocytosis following the introduction of a gluten-free diet.

**Case presentation:**

A 7-year-old white girl presented with fevers and maculopapular rash with a recent history of tonsillitis. Blood tests revealed thrombocytopenia (64×10^9^/L), anemia (80 g/L), hypofibrinogenemia (1 g/L), and hyperferritinemia (71,378 μg/L). A bone marrow revealed evidence of hemophagocytosis, but the results of tests for the genetic or familial-associated hemophagocytic lymphohistiocytosis syndromes were negative. The results of screening tests for known secondary causes were negative. She was diagnosed as having hemophagocytic lymphohistiocytosis and following treatment with the hemophagocytic lymphohistiocytosis-2004 protocol these symptoms, in addition to the biochemical and hematological markers, completely resolved.

She presented again 10 months later with fever, rash, and biochemical abnormalities suggestive of hemophagocytic lymphohistiocytosis. Her tissue transglutaminase was markedly raised and the results of blood tests revealed a genetic susceptibly to coeliac disease in the form of HLA-DQ2 positivity. She commenced a gluten-free diet and there was complete symptomatic and biochemical response without any further chemotherapy. She had further episodic rashes, each associated with the accidental intake of gluten.

**Conclusions:**

This is to the best of our knowledge the first documented case of hemophagocytic lymphohistiocytosis in association with coeliac disease. No other secondary cause found; she initially responded to chemoimmunotherapy specific for hemophagocytic lymphohistiocytosis but relapsed within a few months of cessation of treatment and then achieved complete remission on gluten withdrawal alone.

## Background

Hemophagocytic lymphohistiocytosis (HLH) is a multisystem inflammatory disorder caused by persistent activation of the immune system, particularly macrophages and T cells [1]. Hemophagocytosis by macrophages is the hallmark of this syndrome and is most commonly identified in the bone marrow. Decreased natural killer (NK) cell activity resulting in reduction of cellular cytotoxicity causing persistent antigen presentation and cytokine release leading to macrophage activation is an important disease mechanism [1, 2]. Macrophage activation leads to the secretion of cytokines such as tumor necrosis factor alpha and interleukins (ILs), particularly IL-1 and IL-6, causing further T cell and macrophage activation [1].

The hyperactive immune system causes the classic clinical features of persistent fever, rash, arthralgia and splenomegaly. Laboratory investigations show cytopenia, hypofibrinogenemia, hypertriglyceridemia, and hemophagocytosis [2]. Diagnostic criteria outlined by the Histiocyte Society, based on clinical and laboratory findings, are well described in the literature [2].

## Case presentation

We present the interesting case of a 7-year-old white girl with HLH in association with coeliac disease (CD). She first presented with a 2-week history of fever, associated with tonsillitis, which was not responding to antibiotics. She also had arthralgia and weight loss; an examination revealed a widespread maculopapular rash with no other specific features and no hepatosplenomegaly. She had previously been well, with no significant family history or long-term medications.

Blood tests revealed thrombocytopenia (64×10^9^/L), anemia (80 g/L), liver function derangement (alanine aminotransferase 1038 IU/ml), and hypofibrinogenemia (1 g/L). She had a raised ferritin level (71,378 μg/L), and a bone marrow biopsy showed occasional hemophagocytosis. Her perforin expression was normal by flow cytometry, and cytotoxic T lymphocyte (CTL) and NK cell granule release on stimulation by mitogens was normal. Genotyping of the *PRF* gene encoding perforin protein was wildtype. The result of an autoimmune screen, including rheumatoid factor, anti-neutrophil cytoplasmic antibody, anti-double-stranded DNA, and anti-citrullinated protein antibody was negative. The results of a viral screen including Epstein–Barr virus (EBV), parvovirus, human herpes virus 6, enterovirus, adenovirus, cytomegalovirus (CMV), human immunodeficiency virus, and hepatitis screen were negative.

She commenced treatment on the HLH-2004 protocol, with etoposide, dexamethasone, and ciclosporin given over 40 weeks for a diagnosis of HLH for which no underlying cause was identified. She completed her treatment with a good clinical and biochemical response. Ten months later, she re-presented with a history of fevers, headaches, and lethargy. Blood tests showed high ferritin (6702 μg/L) and lactate dehydrogenase (1002 units/L) levels. She had no cytopenia and a repeat bone marrow aspirate showed no evidence of hemophagocytosis.

Her subsequent clinical course was complicated by recurrent fevers, arthralgia, widespread macular rash, and weight loss. Her ferritin and lactate dehydrogenase levels remained markedly raised and the results of repeat virology investigations were negative. A repeat autoimmune screen revealed markedly raised tissue transglutaminase (tTG) antibodies of 108 Units/ml (normal range 0 to 6.9 Units/ml). A jejunal biopsy was deemed too high risk and a gluten-free diet was urgently commenced. Genetic testing revealed a susceptibility to CD, in the form of human leukocyte antigen (HLA)-DQ2 positivity, and she was therefore diagnosed as having CD.

**Fig. 1:**
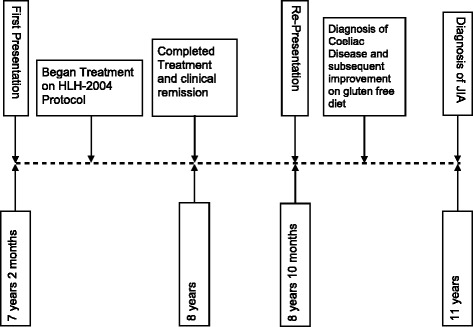
Timeline demonstrating clinical course with concurrent age. *HLH* hemophagocytic lymphohistiocytosis, *JIA* juvenile idiopathic arthritis

She continued a gluten-free diet following which her clinical features improved and her biochemical markers, including tTG antibodies, gradually returned to normal. She had no further recurrences of her HLH symptoms since the introduction of a gluten-free diet. However, she continued to have intermittent episodes of macular rash and arthralgia, particularly associated with accidental ingestion of gluten. Two years subsequent to this she developed symptoms suggestive of juvenile idiopathic arthritis (JIA) without HLH or macrophage activation syndrome (MAS) and received treatment with systemic steroids in addition to methotrexate, with clinical resolution. Her autoimmune profile remained negative. Figure 1 elucidates her clinical course.

## Discussion

HLH has many secondary causes which are well described in the literature, including connective tissue diseases. The morbidity of these syndromes can be significant and accurate diagnosis is important. The recognition of CD-associated HLH has yet to be made. This is the first reported case of HLH secondary to CD, with dramatic improvement of symptoms and biochemical features following the introduction of a gluten-free diet.

An important differential diagnosis of HLH is MAS seen with autoimmune diseases, such as rheumatic fever. Although it can be argued that the hypercytokinemic presentation in this case was as a result of MAS rather than HLH, all the HLH criteria at the time of the original presentation were met and symptoms only resolved on full treatment with standard chemotherapy. In addition, she relapsed a few months after cessation of treatment and her symptoms resolved on gluten withdrawal alone.

However, primary HLH is genetic, commonly autosomal recessive, and specific genes have been identified [1, 2]. In familial HLH, mutations in genes encoding proteins responsible for CTL and NK cell cytotoxicity are present [1, 2]. Treatment of primary HLH by chemotherapy alone invariably leads to relapse and a definitive cure can only be achieved by hemopoietic stem cell transplantation [2]. Secondary HLH occurs when no family history or genetic markers are present and an underlying secondary cause is found [2]. Common secondary causes include: infection with tuberculosis, EBV, CMV, and adenovirus [1, 2]; lymphomas; JIA; and autoimmune diseases [1]. MAS has been reported to be present in 10 % of patients with JIA [3]. Secondary HLH has a high mortality and morbidity in the acute setting; however, following treatment of the acute illness there should be a low risk of recurrence. Similar clinical features can be seen in severe sepsis and viral infections [2]. It is also possible to find hemophagocytosis on a bone marrow aspirate as a reactive feature in acutely unwell patients, and patients with non-hematological disorders [1].

CD is a systemic autoimmune disorder triggered by gluten and characterized by enteropathy and a host of systemic features involving skin, bone, and joints and is strongly associated with the presence of HLA-DQ2 or DQ8 haplotypes [4]. HLH has been described as a presenting feature of enteropathy-associated T cell lymphoma, which in itself is well documented in CD [5].

Two years after resolution of HLH, a diagnosis of seronegative JIA was made and treatment instigated in this patient. It could therefore also be argued that her HLH was secondary to JIA. However, she had no diagnostic features of JIA at first presentation, and demonstrated a good clinical response to standard HLH treatment. Following relapse of her hyperferritinemia she demonstrated complete resolution of symptoms and laboratory findings with the introduction of a gluten-free diet. After further exposure to gluten her symptoms returned. JIA is known to have an association with CD, the latter increasingly being understood as a multisystem disorder with many possible immunological manifestations [4]; however, an association of HLH with response to gluten withdrawal has not previously been described.

## Conclusions

The association of HLH and CD, with clinical response to gluten withdrawal, has not been reported in the literature. This case is noteworthy due to the absence of laboratory features of primary HLH, temporary resolution of symptoms following anti-HLH therapy, rapid relapse upon cessation of HLH therapy, and full clinical and biochemical resolution following a gluten-free diet.

## Abbreviations

CD: Coeliac disease
CMV: Cytomegalovirus
CTL: Cytotoxic T lymphocyte
EBV: Epstein–Barr virus
HLA: Human leukocyte antigen
HLH: Hemophagocytic lymphohistiocytosis
IL: Interleukin
JIA: Juvenile idiopathic arthritis
MAS: Macrophage activation syndrome
NK: Natural killer
tTG: Tissue transglutaminase
